# Association between work ability and work stressors: cross-sectional survey of elderly services and health and social care service employees

**DOI:** 10.1186/s13690-022-00841-2

**Published:** 2022-03-15

**Authors:** Kirsikka Selander, Risto Nikunlaakso, Jaana Laitinen

**Affiliations:** 1 Finnish Institute of Occupational Health, Kuopio, Finland; 2 Finnish Institute of Occupational Health, Helsinki, Finland; 3 Finnish Institute of Occupational Health, Oulu, Finland

**Keywords:** Work ability, Work stressor, Moral distress, Health and social care (HSS) employees

## Abstract

**Background:**

Work in the health and social care services (HSS) is very stressful and sickness absences are high. Nevertheless, little is known about their work stressors and work ability. The first aim of this study is to describe the prevalence of different work stressors and their accumulation among eldercare workers compared to general HSS workers. Second aim is to analyze associations between different work stressors and work ability and thus provide information on factors that are important in enhancing work ability.

**Methods:**

This cross-sectional survey examined HSS employees in Finland in 2020. The response rate was 67% (*N* = 22,502). Descriptive analyses were used to describe the control variables and the differences between the work stressors of general HSS and eldercare employees. After this, multinomial logistic regression analysis revealed the association between work stressors and work ability.

**Results:**

Eldercare employees experienced more often moral distress than HSS employees in general, and this further lowers their work ability. Single work stressors––Karasek’s strain, Siegrist’s ERI, organizational injustice and moral distress––increased the odds of low work ability (OR range 1.4–2.5) in comparison to no work stressors. However, the association with single stressors was roughly one third of that with the accumulation of all four work stressors (OR = 6.8). Thus, the accumulation of several stressors was most harmful for work ability.

**Conclusions:**

This study provides novel information on the accumulation of work stressors in relation to work ability. The results suggest that in order to enhance work ability, HSS organizations should pay more attention to preventing several stressors from accumulating. Eldercare organizations in particular need to develop effective measures for lowering moral distress.

## Background

The ageing of the Finnish population and the retirement of health and social care service (HSS) employees has created an urgent need for professional eldercare givers. At the same time, the young age groups entering work life are considerably smaller. Work in HSS is also demanding. Even though the Finnish Occupational Health Care Act [1] obligates employers to care for the work ability of their employees, sickness absence rates, especially due to mental disorders, are high and even rising in Finland [2, 3]. The COVID-19 pandemic has further aggravated psychological distress [4, 5]. To guarantee the quality of eldercare, HSS work needs to seem more attractive for new employees and it must promote sustainable working careers. Improving the well-being of HSS workers and taking measures to support work ability at the early stages of development of work disability can enhance the attractiveness of HSS, prevent work disability, and promote return from sick leave [6–8]. However, more information on work stressors is needed for developing new strategies and ways to enhance work ability.

The concept of work ability was developed in the 1980s by Ilmarinen and colleagues [9]. It is defined as a balance between personal resources and job demands [10]. Strong predictors of work disability are age, self-rated health, number of sickness absences, socioeconomic position, chronic illnesses, sleep problems and body mass index [11]. Less is known of the effect of work stressors on work ability, although they affect both the individual health and the demands of the work [12, 13]. In this study we concentrate on the psychosocial stress models, including Karasek’s demand-control model [14], Siegrist’s Effort-Reward imbalance (ERI) model [15], and Moorman’s organizational injustice model [16]. High job demands combined with low control (Karasek’s model), effort-reward imbalance (Siegrist’s model), and organizational injustice (Moorman’s model) – especially when co-occurring with effort-reward imbalance – have been suggested to be most detrimental to employee health [17–19]. Empirical studies have linked Karasek’s strain, Siegrist’s ERI and organizational injustice also to reduced work ability [20–24].

The basic assumption of Karasek’s model is that high demands combined with low control at work leads to work stress or other negative health risks [14]. Siegrist, in turn, assumes that negative health outcomes result from an imbalance between the efforts of the employee at work and the rewards (e.g. wages, career prospects, esteem and job security) they receive [15]. Moorman’s organizational injustice theory assumes that negative outcomes are due to employees’ perceptions of unfair treatment at the workplace. These may be linked to unfairness in the decision-making process, feelings of being mistreated by one’s supervisor, or unfairness of exchange [16].

Recent studies suggest that more important than the existence of a single stressor is the accumulation of several stressors [25, 26]. Dragano and colleagues [25] showed that a combination of Karasek’s strain and Siegrist’s ERI was more likely to result in coronary heart disease than these stressors separately. Juvani and her colleagues [26] found that a combination of Karasek’s strain, Siegrist’s ERI and organizational injustice increased the risk of work disability due to depression more than these stressors alone or in pairs. The accumulation of work stressors is still a largely unexplored topic, however. To our knowledge, no previous publications analyzing the associations between accumulation of work stressors and low work ability exist.

In addition to traditional work stressors, moral distress is a significant source of strain among HSS employees [27–29] and thus it is considered as the fourth work stressor. Even though different conceptualizations exist, moral distress is mostly understood to arise from external obstacles that prevent employees from acting in accordance with their ethical principles [30, 31]. For example, employees may experience moral distress because they are unable to offer sufficiently high quality of care due to the fast pace of their work. Based on our preliminary analyses, the prevalence of moral distress is nearly twofold in eldercare compared to that in other HSS work. Previous empirical studies have associated moral distress with, for example, decreased job satisfaction, increased burnout and turnover intentions [29]. There is, however, a gap in the knowledge on the effects of moral distress on work ability.

This article has three aims. First, our practical aim is to analyze eldercare workers and compare them with general HSS workers: to describe the prevalence of different work stressors and their accumulation. Second, we aim to broaden the theoretical understanding of the detrimental accumulation of work stressors among HSS workers by including moral distress. Finally, we aim to increase the understanding of the factors associated with reduced work ability by analyzing which combinations of work stressors are the most detrimental. This provides information on the aspects of work in which to intervene when enhancing the work ability of HSS employees before their situation deteriorates and they retire prematurely.

## Methods

### Data

The data for this study were obtained using a cross-sectional survey conducted among HSS employees. The data covered all HSS employees who were actively working in nine Finnish public organizations between 27.10.2020 and 30.11.2020, except for the employees who were on parental, sick or study leave. Of the invited employees 24,459 responded (response rate 67%) and 92% gave their consent to use the data for research (*N* = 22,502; employees without research consent were left out of the analysis). Employees who gave research consent were further classified into general HSS employees (*N* = 18,155) and those who work in close contact with elderly people (*N* = 4,347). Eldercare included all work units in which work involves close contact with the elderly, including immediate superiors. Administrative work, top management and all other work units were included in the general HSS. Classification was based on work unit titles and later confirmed by the contact persons in the customer organizations. The study was approved by the ethical board of the Finnish Institute of Occupational Health. Participation in the survey was voluntary and consent to use the responses for scientific research was requested in the questionnaire.

### Measures

In the analysis, we used self-reported measures of work ability and work stressors, obtained by a survey. Rather than analyzing objective work ability assessed by health professionals, we sought to understand how work stressors influence employees’ perceptions of work ability. Thus, as an outcome measure, we used the first question of the Work Ability Index developed by Finnish researchers [32, 33]: current work ability compared to lifetime best, which is a valid indicator of sickness absences and premature retirement [6, 8]. Work ability was evaluated on a scale of 0 to 10. For the analysis, we chose the lowest decile as low work ability.

Work stressors included Karasek’s strain, Siegrist’s ERI, organizational injustice, and ethical strain. Karasek’s strain, i.e., job demands and control, were measured using four items derived from the Job Content Questionnaire [14]. Job demands were measured by two statements: “I am required to do an unreasonable amount of work” and “I don’t have enough time to get my work done” (scales inverted). Job control was also measured by two questions: “My job involves a lot of similar repetitive tasks”, “I have lot of say in my own work” (scale inverted). The response scale was a five-level Likert-type scale (1 = strongly agree to 5 = strongly disagree). Karasek’s strain was calculated by subtracting the mean of job control from job demands [34].

Siegrist’s ERI were measured using a short four-item proxy ERI measure. Effort was measured by asking one question: “How much do you feel you invest in your job in terms of skill and energy?” and rewards by asking three questions: “How much do you feel you get in return for work in terms of income and job benefits?”, “How much do you feel you get in return for work in terms of recognition and prestige?”, “How much do you feel you get in return for work in terms of personal satisfaction?”. The response scale was a five-point Likert scale (1 = very much to 5 = not at all). The ERI score was calculated by dividing the effort score by the mean of the reward scores [35]. Since the ERI score and Karasek’s strain are based on division and subtracting we were unable to provide a Cronbach’s alpha for them.

To measure organizational injustice, we used the modified version of Moorman’s organizational justice [16] scale, excluding the last dimension of unfairness of exchange. Five statements measured procedural justice: “Decisions made are consistent (the rules are the same for everyone)”, “Effects of decisions are monitored and communicated “, “Additional information on the grounds for decisions is available if desired”, “Decisions are made based on right information”, “Failed decisions can be revoked or changed” and four items measured relational justice: “My supervisor’s personal preferences do not interfere with his/her decisions”, “My supervisor treats his/her subordinates kindly and attentively”, “My supervisor respects employee rights”, “My supervisor can be trusted”. The response scale was a five-point Likert scale (1 = totally agree to 5 = totally disagree). The organizational injustice score was calculated as the mean of these items (Cronbach’s alpha = 0.91).

Moral distress was measured by asking three questions: “How often do you have to consider ethically challenging situations in your work?”, “How often do you have to act against rules and norms?”, and “How often do you have to act against your own values?”. The response scale was a five-point Likert-type scale (1 = never to 5 = daily). The moral distress score was calculated as the mean of these items (Cronbach’s alpha = 0.78).

To analyze the accumulation of work stressors, we combined Karasek’s strain, Siegrist’s ERI, organizational injustice, and moral distress. There was no substantial overlap between the work stressors (all correlation coefficients < 0.45). First, we set the highest quartile of Karasek’s strain, Siegrist’s ERI, organizational injustice, and moral distress as high stressors, and then set the remaining three quartiles as a reference category. After this, we combined these with a 16-category variable that indicated all the possible combinations of the four work stressors (see Table 1). Each respondent belonged to one category only.

**Table 1:** Participant characteristics in general HSS and eldercare

	General HSS	Eldercare	
Characteristics	N (%)	N (%)	*p*-value
All participants	18,155 (80.7%)	4347 (19.3%)	
Gender			
Female	15,399 (84.8%)	4153 (95.5%)	
Males	2756 (15.2%)	194 (4.5%)	< 0.001
Age (years)			
< 30	2207 (12.2%)	578 (13.3%)	
30–39	4331 (23.9%)	767 (17.6%)	
40–49	4744 (26.1%)	1047 (24.1%)	
50–59	5148 (28.4%)	1461 (33.6%)	
> 60	1725 (9.5%)	494 (11.4%)	< 0.001
Supervisor			
Yes	1590 (8.8%)	177 (4.1%)	
No	16,497 (91.2%)	4156 (95.9%)	< 0.001
Occupation			
Practical nurse	1801 (10.8%)	3015 (71.8%)	
Nurse	6379 (38.3%)	689 (16.4%)	
Others	8471 (50.9%)	496 (11.8%)	< 0.001

*P*-values are based on χ^2^-test

The covariates used in the analyses included work unit category (general HSS, elderly care), gender (males/females), supervisory position (yes/no), age as a categorical variable (< 30, 30–39, 40–49, 50–59, > 60) and occupation (practical nurse, nurse, other). Furthermore, we adjusted our parameters by taking perceived health into account. The respondents were asked to assess how they perceived their health: good, fairly good, average, fairly poor, or poor. In the analysis, we treated perceived health as a continuous variable. Even though perceived health shares conceptual similarity with work ability, these concepts do not overlap (correlation coefficient = 0.40). Perceived health and individual lifestyle variables such as physical activity and overweight have previously been associated with work ability [36, 37], but also with perceived job strain [12, 13]. Thus, as perceived health might mediate or confound the association between strain and work ability, it needs to be considered.

### Statistical analysis

We used descriptive and multivariate statistics to analyze the data. First, we used descriptive statistics and the chi-square test to identify statistically significant differences between the control variables of the general HSS and the eldercare employees. Next, we used multinomial logistic regression to analyze the associations between work stressors and work ability. Logistic regression analysis was conducted stepwise using the enter method, thus showing how connections change after new variables are entered into the model. In the first step, we included only covariates (excluding perceived health), showing the initial stage between the general HSS and eldercare employees. In the second step, we added work stressors, and in the final step, perceived health. Perceived health was included in the final step instead of being added to the other covariates because this enabled us to see whether it confounded the association between work stressors and work ability.

## Results

A total of 22,502 responded to the survey, 81% of whom worked in general HSS and 19% in eldercare (see Table 1). Most of the employees in general HSS were female (85%), aged 50–59 (28%) and worked in non-supervisory positions (9%), and in eldercare, these proportions were even higher than in general HSS. In eldercare, most employees worked as practical nurses (72%), whereas in general HSS most respondents were in professions other than nursing (51%). (See Table 1.)

Compared to general HSS, in eldercare, the proportion of employees who perceived their health as “good” was lower (31% versus 39%), and the proportion reporting low work ability (12% versus 9%) was higher. In addition, the proportion that reported having work stressors was higher. In general HSS, nearly half of the employees reported that they were not exposed to any of the stressors (44%), whereas in eldercare, the corresponding proportion was one third (32%; see Table 2). When we looked at the prevalence of one stressor, we observed that the proportion of those with Karasek’s strain and particularly those with moral distress was greater in eldercare than in general HSS. Siegrist’s strain and organizational injustice, in turn, were more prevalent in general HSS. We were also able to see that the accumulation of moral distress and one, two or three other work stressors was more prevalent in eldercare than in general HSS, as was the accumulation of four stressors.

**Table 2:** Accumulation of work stressors

Work stressor					Observed percentage of work stressors	
Number of stressors	Karasek's strain	Siegrist's ERI	Organizational injustice	Moral distress	General HSS	Eldercare
0	-	-	-	-	44.0%	32.0%
1	+	-	-	-	3.5%	4.2%
1	-	+	-	-	5.8%	5.1%
1	-	-	+	-	9.7%	4.5%
1	-	-	-	+	9.5%	18.7%
2	+	+	-	-	2.1%	2.3%
2	+	-	+	-	2.6%	1.4%
2	+	-	-	+	1.8%	4.6%
2	-	+	+	-	3.4%	1.6%
2	-	+	-	+	1.9%	4.3%
2	-	-	+	+	3.9%	4.9%
3	+	+	+	-	3.3%	1.8%
3	+	+	-	+	1.5%	4.3%
3	+	-	+	+	1.8%	2.7%
3	-	+	+	+	1.9%	2.3%
4	+	+	+	+	3.3%	5.6%

### Relation of work ability to work stressors

Table 3 shows the associations between different work stressors and low work ability. In the first step, the odds ratio (OR = 1.17; 95% CI = 1.02–1.34, *p* = 0.03) was higher in eldercare than in general HSS, even when sex, age, supervisory position and occupation were controlled for. However, these differences disappeared in the second step, after controlling for work stressors (OR = 1.09; CI = 0.95–1.26, *p* = 0.28). This indicates that employees in eldercare encounter more work stressors, especially moral distress, and that these stressors accumulate (see Table 2), which is why they more often report reduced work ability than employees in general HSS.

**Table 3:** Association between work stressors and low work ability

	Step1	Step2	Step3
General HSS (ref.)			
Eldercare	1.17 (1.02–1.34)*	1.09 (0.95–1.26)	0.98 (0.84–1.15)
Male (ref.)			
Female	1.12 (0.96–1.30)	9.99 (0.85–1.16)	0.93 (0.78–1.11)
Age (ref. < 30)			
30–39	0.92 (0.76–1.11)	0.89 (0.73–1.08)	1.87 (1.49–2.34)***
40–49	0.84 (0.71–1.00)	0.78 (0.65–0.93)**	1.41 (1.15–1.73)**
50–59	0.77 (0.64–0.91)**	0.73 (0.61–0.87)**	1.11 (0.90–1.35)
> 60	0.97 (0.83–1.15)	0.94 (0.79–1.11)	1.01 (0.84–1.22)
Supervisor (ref.yes)			
No	1.67 (1.30–2.14)***	1.51 (1.17–1.95)**	1.16 (0.87–1.55)
Occupation (ref. Other)			
Practical nurse	1.32 (1.14–1.52)***	1.08 (0.93–1.26)	1.11 (0.94–1.32)
Nurse	1.35 (1.21–1.51)***	0.99 (0.88–1.11)	1.23 (1.08–1.40)**
Work stressors (ref. = none)			
Karasek's strain only		3.21 (2.53–4.07)***	2.45 (1.90–3.23)***
Siegrist's ERI only		2.56 (2.07–3.17)***	1.82 (1.44–2.31)***
Organizational injustice only		1.90 (1.55–2.32)***	1.51 (1.21–1.88)***
Moral distress only		1.56 (1.28–1.89)***	1.36 (1.11–1.68)**
Karasek + ERI		4.95 (3.81–6.42)***	3.37 (2.51–4.53)***
Karasek + injustice		4.61 (3.57–5.95)***	3.26 (2.44–4.34)***
Karasek + moral distress		3.35 (2.54–4.42)***	2.33 (1.72–3.15)***
Siegrist + injustice		3.39 (2.64–4.36)***	2.45 (1.85–3.24)***
Siegrist + moral distress		2.80 (2.09–3.77)***	1.78 (1.28–2.48)**
Injustice + moral distress		2.51 (1.97–3.20)***	2.00 (1.53–2.61)***
Karasek + ERI + injustice		6.67 (5.38–8.26)***	4.37 (3.43–5.57)***
Karasek + ERI + moral distress		6.08 (4.72–7.83)***	3.71 (2.78–4.94)***
Karasek + injustice + moral distress		5.64 (4.36–7.28)***	3.68 (2.76–4.94)***
ERI + injustice + moral distress		5.72 (4.41–7.41)***	3.48 (2.59–4.69)***
Karasek + ERI + injustice + moral distress		10.26 (8.51–12.36)***	6.79 (5.48–8.42)***
Perceived health			4.71 (4.39–5.04)***
Model summary:			
Nagelkerke R^2^	0.01	0.11	0.35
χ^2^ (df)	114.03(9)***	1069.5(24)***	3691.4(25)***
Change in χ^2^ (df)	114.0(9)***	955.5(15)***	2621.9(1)***
N	20,303	20,303	20,303

^***^p < 0.001, **p < 0.01, *p < 0.05

As Table 3 shows, all work stressors reduced work ability, and the accumulation of several stressors was especially harmful. Before interpreting these, however, it is important to consider perceived health, as this weakened the associations between work stressors and work ability, as can be seen in Step 3. Even though the associations between work stressors and work ability became weaker, the accumulation of several stressors was still the most harmful for employees’ work ability (OR = 6.79; CI = 4.39–5.04, *p* < 0.001). Perceived health also had a high OR (OR = 4.71; CI = 4.39–5.04, *p* < 0.001), but based on our analysis, it was not as harmful for work ability as the accumulation of four work stressors. Because ORs of all work stressors and their accumulations decreased from the model 3 to model 4, part of the harmful effect of work stressors on work ability is mediated via perceived health.

**Fig. 1:**
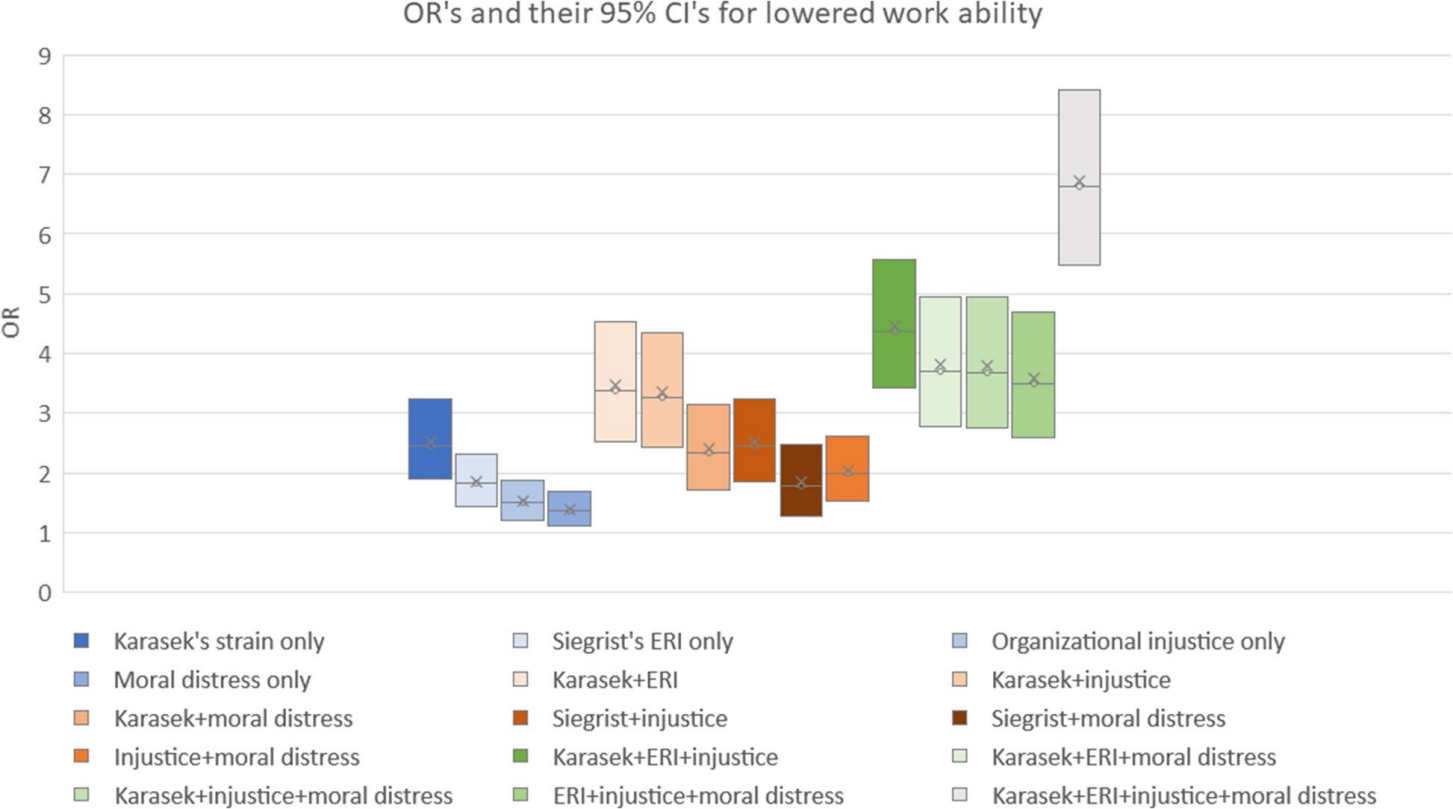
ORs and 95% confidence intervals of the association between single, two, three, and four work stressors with low work ability by multinomial logistic regression; work unit category, sex, age, supervisory position, occupation, and perceived health controlled for

The highest OR for low work ability was for the accumulation of all four stressors (OR = 6.79; CI = 4.39–5.04, *p* < 0.001), followed by perceived health (OR = 4.71; CI = 4.39–5.04, *p* < 0.001) (Fig. 1). Analyses were adjusted for work unit category, gender, age, supervisory position, occupation, and perceived health. In the single stressors, the highest OR was observed for Karasek’s strain (OR = 2.45; CI = 1.90–3.23, *p* < 0.001), but this partially overlapped with other single stressors. For one stressor, the OR for low work ability varied between 1.4 and 2.5 (95% CI 1.1–3.2, *p* < 0.001); for two stressors, between 1.8 and 3.4 (95% CI 1.3–4.5, *p* < 0.001); for three stressors, between 3.5 and 4.4 (95% CI 2.6–5.6, *p* < 0.001); and for four stressors the OR was 6.8 (95% CI 4.4–5.0, *p* < 0.001). The ORs for different combinations of one, two, three, and four work stressors partly overlapped, but a clear trend showed that the more work stressors that accumulated at the same time in the same respondents, the higher was the OR for reduced work ability. (Fig. 1).

## Discussion

The purpose of this study was to provide information on how different work psychosocial stressors accumulate among HSS and eldercare workers, and how this accumulation is associated with work ability. The results can be used for improving the work ability of HSS workers before their situation deteriorates and they retire prematurely. Unlike previous studies, which have mainly focused on associations between single work stressors and work ability [38], we analyzed the accumulation of several work stressors, including moral distress. To our knowledge, no previous studies analyzing moral distress in relation to work ability exist. Based on our results, eldercare employees encounter moral distress more often than other HSS workers, which is associated with lower work ability. Even though single work stressors – Karasek’s strain, Siegrist’s ERI, organizational injustice and moral distress – are positively associated with low work ability, the association with single stressors is roughly one third of the estimate for the accumulation of all four work stressors. Thus, most harmful for work ability is the accumulation of several stressors, and organizations should concentrate on this to enhance the work ability of their employees.

Previous research has shown that the accumulation of Karasek’s strain, Siegrist’s ERI and organizational injustice increases the risk of coronary heart disease [25] and disability pensions [26]. Our results add to those of previous studies, showing that the accumulation of several work stressors is also associated with low work ability and is more harmful than single stressors. These results suggest that the pevention of the accumulation should be key actions to avoid sickness absences as well as early retirement, and intervention studies are needed to prove this hypothesis. The accumulation of psychosocial work stressors in combination with physical strain of the HSS work might further deteriorate the situation considerably [39]. These connections and the profiles of the employees suffering from accumulation of several stressors should be analyzed in more detail, and in longitudinal settings, in the future studies. Future analysis should also focus on different lifestyle variables (e.g. obesity, nutrition and exercising) and years in the current work position, as they are related on employees tolerance to work stressors and thus to work ability.

Our study revealed that moral distress is an important work stressor as such, but also when several work stressors accumulate. This is novel finding, as only a limited amount of research has examined emotional demands (see [38]), such as pupil misbehavior and work ability [40]. To our knowledge, no studies have been conducted on the association between moral demands and work ability, however. Our results show that employees who are exposed to moral distress more often report low work ability than those who are not exposed to it. In addition, the combination of four work stressors was more strongly associated with low work ability than three stressors. Thus, moral distress should be considered when analyzing the work ability of HSS employees.

Moral distress is prevalent among HSS employees [27–29], but our results show that it is even more emphasized in eldercare. Moral distress is one of the key factors that increase the odds of low work ability. As the work pace in eldercare has become more hectic, employees increasingly encounter moral distress, because they are unable to meet the patients’ needs to the extent to which they would like [41, 42]. Thus, organizational-level interventions aiming to prevent under-resourcing as well as individual-level interventions aiming to relieve moral distress could be effective in increasing the work ability of eldercare employees.

Furthermore, our results revealed that eldercare employees experience slightly more Karasek’s strain than general HSS employees. Siegrist’s ERI and organizational injustice, in turn, are more prevalent problems in general HSS than in eldercare. Thus, in addition to moral distress, eldercare organizations should pay more attention to the balance between job demands and resources.

### Strengths and limitations of the study

The main strength of this study is the novel information it provides on the accumulation of several work stressors, including moral distress, and their association with HSS employees’ work ability. These results can be used in formulating hypotheses for intervention studies which promote the work ability of HSS employees. Another strength is that this study focused on eldercare workers, who, to our knowledge, are seldom a target group in work ability studies. Our dataset contained a considerably large number of eldercare respondents (*n* = 4,347).

Our dataset extensively and comprehensively covered Finnish social and health care employees (*N* = 22,502), but it limited us to a cross-sectional design for our study. This prevented causal conclusions and calls for future studies using a longitudinal design. It would be especially interesting to determine the causal relation of the effect of work stressors on work ability. Longitudinal analysis could also show, how Covid-19 pandemic impacts the results. HSS employees are currently under high strain because of the pandemic, and this may exacerbate the adverse effects of work stressors on work ability.

The fact that we used an extensive survey is a strength, but also a limitation. As the survey covered a wide range of topics, we had to use shortened versions of the measurements (e.g. Karasek’s demands), so that response activity would not deteriorate too much. This meant moving away from broadly validated measurements to abbreviated versions. Some of the abbreviated measurements, however, had already been validated or tested elsewhere (see [43] for Siegrist’s ERI). Further, we relied on self-reporting, which can be considered a limitation, as different employees may perceive things differently. Nevertheless, as the results were clear, the measurements can be considered sufficiently valid.

In this study we were particularly interested in the accumulation of several stressors, for which we followed previous studies [25, 26] and concentrated on traditional stress theories. We analyzed whether an imbalance between job demands and resources [14] or efforts and rewards [15] is harmful for work ability. From a practical point of view however, in the future it would be interesting to analyze, using the newer JD-R model proposed by Cadiz and his colleagues [38] for example, which demands, resources, efforts, or rewards are the most harmful to work ability.

## Conclusions

Our study shows that the accumulation of Karasek’s strain, Siegrist’s ERI, organizational injustice, and moral distress is most harmful for work ability, but that these are also strongly associated with low work ability when considered separately, after work unit category, sex, age, supervisory position, occupation, and perceived health are controlled for. Although these associations need to be verified by longitudinal studies, we suggest that organizations should pay more attention to taking preventive measures, especially regarding the accumulation of several work stressors, in order to enhance work ability, promote sustainable working careers, and prevent early retirement. Furthermore, eldercare units should also relieve employees’ moral distress through different strategies to enhance work ability and increase the attractiveness of HSS for employees.

## Data Availability

The data that support the findings of this study are not openly available due to the sensitivity of employee data.

## Abbreviations

HSS: Health and social care service
ERI: Effort-reward imbalance
